# Neutrophils in *Streptococcus suis* Infection: From Host Defense to Pathology

**DOI:** 10.3390/microorganisms9112392

**Published:** 2021-11-20

**Authors:** Marêva Bleuzé, Marcelo Gottschalk, Mariela Segura

**Affiliations:** Research Group on Infectious Diseases in Production Animals (GREMIP) and Swine and Poultry Infectious Diseases Research Center (CRIPA), Faculty of Veterinary Medicine, University of Montreal, Saint-Hyacinthe, QC J2S 2M2, Canada; mareva.bleuze@umontreal.ca (M.B.); marcelo.gottschalk@umontreal.ca (M.G.)

**Keywords:** *Streptococcus suis*, neutrophils, innate immunity, virulence factors

## Abstract

*Streptococcus suis* is a swine pathogen and zoonotic agent responsible for economic losses to the porcine industry. Infected animals may develop meningitis, arthritis, endocarditis, sepsis and/or sudden death. The pathogenesis of the infection implies that bacteria breach mucosal host barriers and reach the bloodstream, where they escape immune-surveillance mechanisms and spread throughout the organism. The clinical manifestations are mainly the consequence of an exacerbated inflammation, defined by an exaggerated production of cytokines and recruitment of immune cells. Among them, neutrophils arrive first in contact with the pathogens to combat the infection. Neutrophils initiate and maintain inflammation, by producing cytokines and deploying their arsenal of antimicrobial mechanisms. Furthermore, neutrophilic leukocytosis characterizes *S. suis* infection, and lesions of infected subjects contain a large number of neutrophils. Therefore, this cell type may play a role in host defense and/or in the exacerbated inflammation. Nevertheless, a limited number of studies addressed the role or functions of neutrophils in the context of *S. suis* infection. In this review, we will explore the literature about *S. suis* and neutrophils, from their interaction at a cellular level, to the roles and behaviors of neutrophils in the infected host in vivo.

## 1. Introduction

*Streptococcus suis* is a porcine bacterial pathogen responsible for important economic losses to the swine industry worldwide [1]. This infection induces severe clinical conditions, including septicemia with sudden death, meningitis, arthritis and endocarditis, mainly in weaned piglets. This zoonotic agent also represents a threat for human health causing mainly meningitis and septic shock [2,3,4]. *S. suis* is classified in 29 serotypes based on its capsular polysaccharide (CPS) antigenicity. The serotype 2 is the most prevalent and associated with disease in swine and humans worldwide. The serotype 9 has more recently emerged in some European countries amongst the most prevalent serotypes in swine clinical cases [5]. Besides serotyping, strains can be genotyped using multilocus sequence typing (MLST). This technique allows strain classification in sequence types (ST) of which distribution appears to be geographical: ST1 strains are mainly isolated in Europe, Asia, Africa and South America, while ST7 strains emerged in China where they caused two major human outbreaks in 1998 and 2005. In North America, strains recovered from swine clinical cases predominately belong to ST28 and ST25 [4,5].

In pigs, *S. suis* colonizes the upper respiratory tract and may breach the mucosal barrier to reach the bloodstream. Infection through the intestine after oral infection has also been suggested, although not yet proved [6]. In humans, bacteria enter via skin lesions or after raw/uncooked pork consumption. Once in the bloodstream, bacteria survive clearance by the immune system and disseminate throughout the body. *S. suis* invasion induces immune system activation (i.e., inflammation), including cytokine production and immune cell recruitment. Although efficient pathogen clearance requires inflammation, its exacerbation in *S. suis* infection leads to host damage contributing to clinical manifestations [7].

What could explain such effervescence of the immune system? For now, the answer remains partially undiscovered, but many studies have addressed this question to provide a better understanding of the role of different immune cell types in the pathogenesis of the disease caused by *S. suis*. The role of neutrophils, an important effector cell type among the first recruited during the infection, has particularly interested researchers since neutrophil infiltration characterizes *S. suis*-induced lesions [7,8,9,10,11].

Neutrophils (also called polymorphonuclear neutrophils (PMNs) in various publications) are immune cells belonging to the family of granulocytes, which is composed of neutrophils, eosinophils and basophils. Neutrophils represent the majority of blood leukocytes and can quickly be mobilized to the site of infection where they initiate and maintain inflammation [12,13]. These cells possess vast effector properties, and their intervention is usually enough to clear minor infections. Neutrophils efficiently phagocyte pathogens either under non-opsonic conditions or mediated by antibody and/or complement opsonization. Phagocytosis is often associated with two other bactericidal mechanisms: the degranulation and the production of reactive oxygen species (ROS). Degranulation consists of the release of the content (proteases, lysozymes, etc.) of cytosolic granules by their fusion to the plasmatic membrane. The ROS are the different forms of oxygen molecule-derivates, which are extremely reactive [14]. Both degranulation and ROS production can occur: (a) inside the phagosome, where concentration of these molecules allows an efficient pathogen killing; and/or (b) outside of the cell, where molecules can target microbes of the close environment. However, in the latter case, granular molecules and ROS toxicity do not spare environing host tissues leading to potential collateral damage.

Besides these mechanisms, Brinkman et al. [15] reported that neutrophils release chromatin and granule-contained proteins. They named these structures neutrophil extracellular traps (NETs) and demonstrated that they entrap and kill pathogens [15]. This phenomenon raised a new interest for neutrophils in the last decade and researchers studied the presence of NETs and their roles in immune system defense against pathogens [16].

Neutrophils not only act as effector cells, but they also participate in regulation of the immune response through cytokine production. Cytokines allow communication between cells and coordinate the immune response in a context of infection [17]. Neutrophils produce less mRNA and proteins than other cells (monocytes/macrophages or lymphocytes), but they are massively recruited at the infection site, and neutrophil-derived cytokines could be of significance in immunomodulation [17]. Cytokine production results in cell recruitment and activation, and their modulation determine an efficient immune protection.

Usually, fine control of neutrophils maintains a proportionate reaction of the immune system towards infection. Uncontrolled neutrophil production and recruitment may worsen outcome in a certain number of infections [18,19]. Understanding the role of neutrophils could help with picturing the initiation of the disease and provide a better characterization of their effect on the outcome of *S. suis* infection. Are neutrophils required for bacterial clearance by vigorously combating the pathogen, or do they participate in the exacerbation of the inflammation that lead to host damage? To untangle our knowledge about the importance of neutrophils in the infection and how they interact with *S. suis*, this review aims to carefully analyze and compare the in vitro and in vivo studies that evaluated these aspects.

## 2. General Analysis of Published Literature

### 2.1. Animal Models or Species-Origin of the Neutrophils

**Figure 1:**
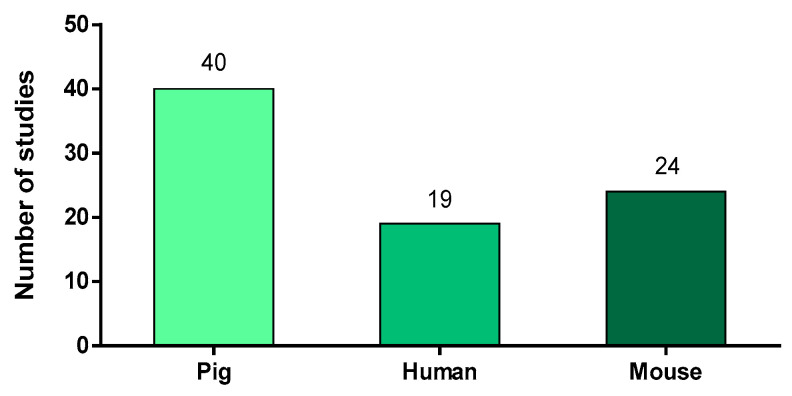
Distribution per target species of published studies addressing *S. suis* interactions with neutrophils or their role during *S. suis* infections. If more than one animal model was used in a study, it appears separately in the graphics. Source: PubMed^®^ (from October 1987 to August 2021).

To the best of our knowledge and based on a PubMed^®^ search, 76 articles referred to neutrophils in the context of *S. suis* infections. In these studies, the authors used three different animal models: pigs, humans and mice (Figure 1). Of note, some studies used more than one animal model. As expected for the study of a porcine pathogen, the majority of the 76 studies (53%) described pigs as a source of cells or as an infection model. Neutrophil purification from their blood is relatively easy to proceed, giving a high yield of cells and limiting the number of animals used. However, the use of this model for in vivo experiments represents an important cost and the infection is not easy to reproduce under laboratory conditions, especially for serotypes other than 2 [4]. Because *S. suis* is part of the normal pig microbiota, animals naturally present antibodies against *S. suis*, with titers increasing with age [20,21,22]. This natural presence of potentially opsonizing antibodies complicates result interpretation. The lack of porcine immunological tools may also limit the study of neutrophils in this model.

Because of the points aforementioned, researchers also frequently used the mouse model for in vivo studies. Although not a natural host for *S. suis*, infected mice develop systemic and brain disease similar to those observed in pigs [7]. The mouse model benefits from a variety of immunological tools available for the study of neutrophils. When used for in vitro studies, mouse neutrophils are often purified from the bone marrow. Indeed, the small amount of blood available per mouse limits the yield of neutrophils obtained. Mouse bone marrow contains both mature and immature neutrophils and this must be taken into account to reach the correct conclusions (for example, when evaluating lifespan of cells under stimulation).

*S. suis* infects not only pigs, but also humans in contact with pigs or their by-products. Therefore, 19 studies evaluated the interactions of human neutrophils with *S. suis* strains, mostly recovered during the two major human outbreaks that occurred in China in 1998 and 2005 [23]. In these studies, neutrophils were isolated from the blood of healthy donors. As human cells are very well studied, the tools for the characterization of neutrophil responses are largely available.

Transposition of conclusions from one animal model to another is tempting but should be carefully addressed due to the many differences existing between species. Porcine, murine and human neutrophils differ in several aspects that could potentially influence the results. First, the three species present different neutrophil proportions in blood: they represent 20–70% of the leucocytes in pigs [24,25,26,27] and 50–70% of leukocytes in humans [28], but account for only 10–30% of mouse total blood leukocytes [24,28]. Interestingly, swine neutrophils weakly respond to phorbol 12- myristate 13-acetate (PMA), calcium ionophore (A23187) and their response to *N*-formyl-methionyl-leucyl-phenylalanine stimulation is controversial [29,30,31,32,33]. On the other hand, those molecules strongly activate mouse and human neutrophils. The three animal models also present different types and amounts of antimicrobial proteins. For instance, human neutrophils are important sources of defensins that are not expressed by cells from mouse origin [28,34]. Pigs seem to express only one β-defensin; however, they possess a wider repertoire of cathelicidins than any other species (around 11 different members of this family) [35]. Finally, the morphology, the granularity, the surface molecule expression and chemotactic properties greatly vary between swine, mouse and/or human neutrophils [33,36]. These differences in neutrophil properties may affect the observations made and thus limit our interpretations to the sole model studied.

### 2.2. S. suis Serotypes and Sequence Types

**Figure 2:**
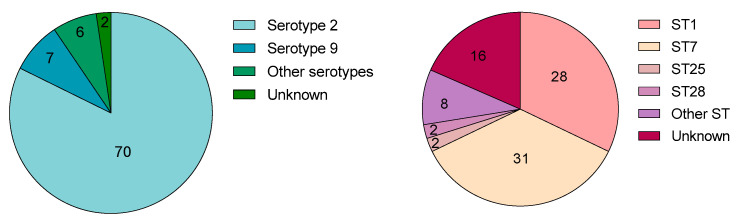
Distribution per *S. suis* serotypes and/or multilocus sequence typing-based sequence types (ST) of strains used to address *S. suis* interactions with neutrophils or their role during *S. suis* infections. If more than one serotype or ST was described in the same study, it appears separately in the graphics. Source: PubMed^®^ (from October 1987 to August 2021).

Of the 29 described serotypes, serotypes 1–9 and 14 are the most frequently isolated from diseased pigs [37,38]. The 76 studies interested in neutrophil behavior during *S. suis* infection mainly used serotype 2 strains (92%), which is the most important serotype worldwide (for both pigs and humans) and the most frequently studied (Figure 2). Few articles studied serotype 9, particularly frequent in Europe and the second most important in the world [5]. Some studies evaluated the potential for a vaccine to induce opsonizing antibodies against both serotypes 2 and 9, mediating an efficient neutrophil-dependent killing of *S. suis* [39,40]. Other studies addressed mechanistic differences existing between the two serotypes, such as how they differently induce lesions, influence oxidative burst or survive killing by neutrophils [9,41,42]. Importantly, diverse serotypes may affect immune responses differently, as demonstrated with neutrophils [42] and other cell types [43]. More comparative studies are thus required to understand the influence of the serotype involved on neutrophil responses.

When STs were considered among the studies interested in neutrophils, the most commonly used strains belonged to ST1 and ST7 (Figure 2). ST1 strains predominate in Eurasia, Africa and South America, while ST7 strains are endemic to China and caused the outbreaks in 1998 and 2005 [5]. Few studies used ST28 and ST25 strains, which are predominantly isolated in Canada and USA [44,45,46]. Once again, different STs induce different immunological responses as demonstrated in vitro [47,48,49,50] and in vivo [51], reflecting the important heterogeneity of *S. suis*.

Although it seems logical to study the “classical” strains of *S. suis* (serotype 2, ST1, ST7), the number of studies available for other serotypes or STs is still limited. The use of a greater diversity of strains would provide a better overview of *S. suis* immunopathogenesis and target common mechanisms.

### 2.3. Purity and Viability of Neutrophils

#### 2.3.1. Isolation Methods and Influence on the Purity of Neutrophils

The majority of the articles reviewed herein used an in vitro approach to characterize neutrophil interactions with *S. suis*. However, neutrophils are short living cells and very sensitive to activation [52]. Therefore, the isolation methods must limit cell activation. To maintain neutrophils as alive and inactivated, they should be freshly isolated using endotoxin-free materials and reagents, and experimental procedures must be conducted as quickly as possible after isolation [53,54]. One classical method for neutrophil purification consists of density gradient centrifugation. Blood or cell suspensions are layered on a continuous or discontinuous gradient density solution, and centrifugation allows cell separation based on their density [53]. This common approach separates granulocytes in a rapid manner with a limited activation of the cells. However, neutrophils cannot be separated from other granulocytes since basophils, eosinophils and neutrophils have a similar density [53,55]. Eosinophils represent the main contaminant cell type in neutrophil suspensions, but it was demonstrated that they have very little effect on gene expression and cytokine production when they contaminate neutrophil suspensions [55,56]. Another limitation of the gradient sedimentation is that a population of low density granulocytes migrate through the gradient the same way as mononuclear cells and researchers must take into account the exclusion of that population of neutrophils in their analyses [57].

**Figure 3:**
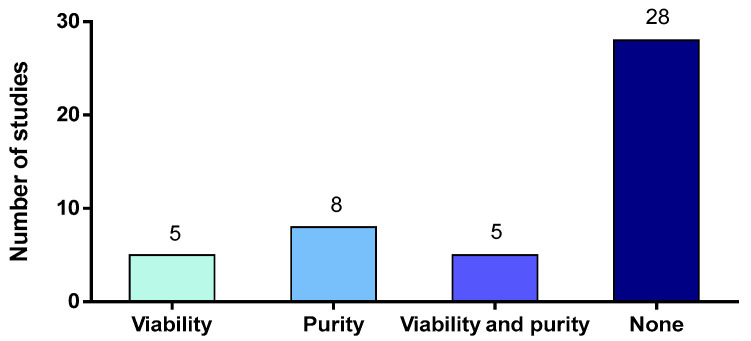
Number of studies evaluating neutrophil purity, viability or both among those addressing *S. suis* in vitro interactions with these cells. Source: PubMed^®^ (from October 1987 to August 2021).

Density gradient separation is often coupled with dextran sedimentation, which allows a separation between red blood cells and leukocytes. However, Quach and Ferrante [58] reported that dextran sedimentation induces neutrophil activation by monocytes. In some studies, dextran alone was used for cell isolation, based on an early study in 1977 [59]. However, this method may provide a low purity of neutrophils since it does not allow acceptable leukocyte separation. Using this method, one study on *S. suis* interactions with neutrophils reported 75% of purity, which was the least pure suspension of all the articles on this pathogen that measured it [45] (Figure 3). This indicates the necessity of density gradient centrifugation for the isolation of neutrophils.

Very pure neutrophil suspensions (up to 99%) can be obtained from human or mouse, using well-described techniques. First, a positive enrichment of neutrophils can be achieved using a fluorescence-activated cell sorting (FACS), but it potentially activates the neutrophils [60]. Some immunomagnetic negative selection kits are commercially available, and it is possible to design in-house antibody panels, as described by Hasenberg et al. [61]. Of note, despite of the high purity provided by these techniques, they remain expensive, and the yield of neutrophils is low. On the other hand, fewer tools are available for pigs, challenging research with this animal model.

Working with a highly pure cell suspension is the basis of in vitro studies, although no purification method guarantees 100% purity. As evocated earlier, eosinophils often contaminate neutrophil suspensions. Assuming that they do not contribute significantly to the measured parameters, most studies applied the nomenclature neutrophils (or PMNs) instead of granulocytes to qualify these mixed cell suspensions, with the exception of three studies that kept the term granulocytes [42,62,63]. Besides eosinophil contamination, a study assessed the importance of cell purity when isolating human neutrophils [56]. The authors claimed that a purity below 99% increases the risk of contamination with monocytes and other cells (including dendritic cells). Because neutrophils possess small amounts of RNA and produce few cytokines [64], this contamination could drastically tamper cytokine production by neutrophils. Contaminating cells may also interfere with other processes, since studies described production of extracellular traps (ETs) by macrophages [65] and other cell types [66] as well as ROS production by several cellular sources [46,67]. In addition, it has been proposed that bacterial lipopolysaccharide (LPS) induces a prolonged survival of neutrophils; however, this phenomenon was indeed related to LPS activation of contaminating monocytes and was abolished when they were depleted [68]. Thus, measuring neutrophil purity after isolation may be crucial for correct interpretation of neutrophil behaviors.

Among the 46 articles studying in vitro activation of neutrophils by *S. suis*, only 13 studies (28%) measured neutrophil purity after isolation [44,45,54,62,69,70,71,72,73,74,75,76,77] (Figure 3). A diversity of methods were used to assess cell purity: Giemsa/Wright’s stain [44,73,74], Hoechst stain (microscopy) [71,72] or flow cytometry using different markers (for example, Ly6G+, CD11b+) [54,75,76,77]. However, in these studies, the purity was comprised between 75% and 97% and the high risk of contaminating cells probably compromised the interpretation of the results when the purity was low.

#### 2.3.2. Neutrophil Viability

Neutrophils are fragile cells unable to divide and should be used freshly after isolation to ensure live and functional cells. For human neutrophils, the lifespan in vitro seems to be less than 20 h [78]. The half-life of mouse neutrophils purified from blood reaches 6 h, while those from bone marrow survive more than 12 h [79]. In pigs, a recent report concluded that a 24 h storage of porcine blood led to a decrease in the number of viable neutrophils after isolation. In addition, storage seems to activate neutrophils since they showed enhanced microbicidal activities [54]. Thus, it is very important to measure neutrophil viability after purification—to ensure that the isolation procedure does not affect the cells, and after in vitro stimulation—to ensure non-cytotoxic conditions. Of the 46 studies in vitro using purified neutrophils, only 10 studies measured viability after isolation or after stimulation [44,54,62,73,74,80,81,82,83,84] but none of them measured both (Figure 3). When evaluated, viability was measured via Trypan Blue exclusion or flow cytometry. Surprisingly, this review reveals that, in many publications, neutrophil viability was not estimated, although this feature determines the correct interpretation of the results.

## 3. In Vitro Functionality

As mentioned, a majority of studies characterized *S. suis* interactions with neutrophils in vitro. Thus, different neutrophil functions were tested after *S. suis* stimulation.

### 3.1. Killing of S. suis by Neutrophils: Still a Controversy

Neutrophils are the first line of defense during an infection. Endowed with many killing mechanisms, these powerful effector cells can clear a pathogen threat even before the induction of an adaptive response. The global antimicrobial effect of the cells comprises phagocytosis but also the extracellular toxic effect of granular proteins, ROS and NET-associated proteins. To assess *S. suis* killing by neutrophils, the simplest method consists of comparing the growth of the bacteria in the presence and in the absence of neutrophils. However, levels of killing are hard to compare amongst the studies due to variations in technical details (multiplicity of infection, percentage of serum, incubation time, etc.). Even the way to calculate *S. suis* killing by neutrophils greatly differed: some studies presented *S. suis* survival factor or percentage of bacterial survival, while others expressed data as percentage of bacterial killing or even raw bacterial count.

Of note, killing is enhanced by opsonizing agents such as antibodies and complement, that can naturally be found in the serum (see below). To prevent interference in a killing test, the serum, being a source of nutrients for the cells in numerous culture media, should be free of *S. suis*-specific antibodies, which is difficult to obtain if working with pigs. If the serum used is inactivated, it should be clearly stated. It should be considered that under in vivo conditions, complement is always present (see below).

Numerous studies have demonstrated that neutrophils fail to eliminate serotype 2 *S. suis* strains [39,40,44,71,80,85,86,87,88,89,90,91,92], although some studies reported important *S. suis* killing by neutrophils (from 40% to 80% of killing) [42,69,80,93,94,95,96,97,98]. As evocated previously, methodological differences may explain this discrepancy.

### 3.2. Phagocytosis: Neutrophils Poorly Phagocytize S. suis

Phagocytosis represents a crucial mechanism for immune cells, particularly professional phagocytes such as macrophages, dendritic cells and neutrophils. A pathogen internalized by the cells will be inside a vesicle called phagosome that later fuses with the lysosome. This second vesicle contains microbicidal enzymes and a very low pH that destroy a variety of microbes [99]. Detection of *S. suis* phagocytosis requires precise methods such as flow cytometry with fluorescent *S. suis* strains [44,100], transmission electronic microscopy (TEM) [44], double immunofluorescence staining [82] or antibiotic-protection assay [82,101]. As *S. suis* can be extracellularly bound to cells [82,102,103], the methods used should discriminate between inside and outside bacteria (double-differential immunofluorescence, use of antibiotics, and appropriate controls). For studies where intracellular and extracellular bacteria were not distinguished, the authors of this review preferred to use the term “association” instead of “phagocytosis” (as it is often misused in the articles). Sometimes, studies evaluate phagocytosis using the actin-polymerization inhibitor cytochalasin D, since it stops intracellular movement such as phagocytosis. However, these results obtained using cytochalasin D should be carefully interpreted since this molecule lacks specificity and may also affect other neutrophil functions such as degranulation [104]. Phagocytosis is also enhanced by the presence of opsonizing agents, such as antibodies and complement found in the serum, and their effect should be considered when analyzing the data obtained (discussed below).

Published in 1998, one of the first articles that aimed to evaluate *S. suis* phagocytosis by neutrophils reported that more than 90% of human granulocytes and monocytes, 77% of swine granulocytes and 67% of swine monocytes were associated with *S. suis* [105]. However, recent studies using more accurate methods revealed that very few bacteria are actually phagocytized by/associated to neutrophils. Lun et al. [100] demonstrated, using two fluorescent strains, that less than 6% of porcine granulocytes contained associated *S. suis*. Another study, using double immunofluorescence staining, showed that *S. suis* is found both outside and inside the transmigrated porcine neutrophils in vitro, although data were non-quantitative [84]. Though phagocytic rates of *S. suis* by neutrophils are low, a study suggested that porcine neutrophils possess higher phagocytic abilities than peripheral blood mononuclear cells, and rapidly kill 80% of the intracellular *S. suis* [82], which is in agreement with other studies carried out with phagocytes that showed that *S. suis* is quickly degraded intracellularly [106,107].

### 3.3. Host Factors Facilitating S. suis Phagocytosis and/or Killing by Neutrophils

As mentioned above, the serum contains opsonizing factors, including the complement and the antibodies, which facilitate bacterial internalization and killing. Nevertheless, few studies evaluated the influence of opsonization on *S. suis* phagocytosis by neutrophils (i.e., using a method able to discriminate between intracellular vs. extracellular bacteria). A complete serum complemented with purified *S. suis*-specific IgG increased neutrophil ability to kill *S. suis* [44]. However, a complete serum alone or a convalescent serum did not [44,82]. Indeed, contradictory studies exist concerning the role of serum or plasma in increasing porcine neutrophil-mediated killing, which might depend on the use of a non-encapsulated *S. suis* mutant [54,108] vs. a wild-type encapsulated serotype 2 strain [93,96]. In an antibiotic protection assay, human neutrophils phagocytized only 0.5 to 1% of opsonized bacteria [101], yet the opsonins involved were not described in the study. With porcine neutrophils or granulocytes, levels of bacterial association in presence of serum (such as heat-inactivated convalescent serum or hyperimmune serum) were either not increased or lower than 20% [42,44,100]. However, TEM revealed that neutrophils internalized IgG-opsonized bacteria (non-quantitatively analyzed) [44]. In absence of IgG, very low levels of *S. suis* association were observed, and were mainly driven by IgM and/or complement activation [42].

It should be noted, that in spite of addition of host factors, such as serum, neutrophil killing of encapsulated wild-type *S. suis* remains in general very low; yet, strain-specific and/or serotype-specific features might affect the overall resistance level [44]. From immunization studies, it has also been evidenced that high levels of vaccine-induced opsonizing antibodies are indeed required to induce neutrophil-mediated killing of encapsulated *S. suis* [39,83,109]. However, this effect is greatly influenced by the antigen used in the vaccine formulation [39,40,88].

In the immune response, cells communicate through circulating molecules, the cytokines, which coordinate cellular response. Cytokines can “prime” neutrophils, turning them into a preactivated state that will maximize their activation if stimulated with a second signal [110]. Only one study evaluated the effect of neutrophil priming by cytokines on *S. suis* killing [44]. GM-CSF, IL-8 and TNF-α enhanced killing abilities of neutrophils while IFN-γ and IL-1β did not. Interestingly, the complete serum improved TNF-α effect on killing but the authors provided no explanation [44].

Another study suggested a role for host DNAse I in *S. suis* killing by neutrophils, particularly in meningitis [108]. Surprisingly, host DNAse I treatment enhanced the killing of a non-encapsulated mutant of *S. suis* by neutrophils in presence of plasma. The authors hypothesized that NETs entrap *S. suis* without killing, and that DNA cleavage by the host DNAse I releases bacteria, allowing neutrophil phagocytosis and killing. The presence of plasma would favor phagocytosis over NETs, while its absence may promote NET release. However, when using an encapsulated *S. suis* strain, this phenomenon of DNase I-mediated enhancement of neutrophil killing was not confirmed. In addition, in their model of porcine blood–cerebrospinal fluid (CSF) barrier in vitro [108], transmigrated neutrophils failed to eliminate wild-type *S. suis* even in the presence of host DNAse I, suggesting that this mechanism would not occur under natural conditions.

### 3.4. S. suis Strategies to Resist Neutrophil-Mediated Phagocytosis and/or Killing

Even facing the most aggressive cells of the immune system, *S. suis* resists killing. Many articles in the field of *S. suis* research aimed to identify and characterize *S. suis* virulence factors, to better understand how *S. suis* goes from a commensal to a pathogen microorganism. Particularly, how invasive strains survive in the blood, a niche full of immune cells—including a large amount of neutrophils.

The main virulence factor responsible for *S. suis* resistance to neutrophil-mediated killing is the CPS [44,71,82,86,87,111], which protects bacteria against phagocytosis [44,54,71,82], limits bacterial adherence to the cells [82], the microbiocidal effect of neutrophil secretions and NET trapping, as further discussed below.

It has been highlighted that the suilysin (SLY)—a hemolytic toxin produced by *S. suis*—participates in the resistance to neutrophil-mediated killing, independently of its cytotoxic effect on the cells [44,82]. This was demonstrated using different approaches including the use of SLY-negative natural strains, SLY-negative mutant strains, SLY inhibitors, and recombinant SLY [44,82]. However, no effect of SLY on *S. suis* association/adherence to porcine granulocytes was observed [82,100]. Other studies suggested that this toxin inhibits neutrophil phagocytosis/killing under opsonic conditions [82,101]. This effect could therefore be linked to complement inhibition by SLY. It was hypothesized that SLY consumes the complement or prevents its deposition at the bacterial surface, but the precise mechanisms remain to be elucidated [44].

Among strategies to resist neutrophil-mediated phagocytosis and/or killing, the two-component regulatory systems (TCSs) allow bacteria to sense environment stimuli and respond by modulating gene expression [112]. Thus, bacteria could enhance expression of genes promoting its resistance to neutrophil-mediated killing. It was the case for some TCSs, including VraSR [85] and the catabolite control protein A (CcpA) [111], that upregulated genes involved in CPS thickness and cell wall synthesis. CcpA senses glucose availability, a nutrient present in the plasma, suggesting that *S. suis* may enrich its CPS in the blood. Other TCSs involved in resistance to killing were also described: SalK/SalR [80] and NisK/NisR [113]. Their genes are localized in a putative pathogenicity island, 89K PAI, specific to highly virulent Chinese strains [80,113], but it remains unclear how they regulate gene expression and how they favor bacterial resistance to neutrophil-mediated killing. Interestingly, Pan et al. [95] described a TCS, named as control of virulence regulator (CovR), that negatively regulates *S. suis* virulence. The Δ*covR* mutant survived more than the wild-type strain to human and porcine neutrophil-mediated killing. Indeed, CovR seemed to reduce hemolytic activity, CPS thickness and limit chain formation [95].

Hui et al. [103] recently identified the bacterial protein HP0487 that promotes *S. suis* adhesion to neutrophils but limits phagocytosis. The property of this protein corroborates the idea that *S. suis* may travel throughout the body attached to leucocytes [114], but the importance of neutrophils in that phenomenon needs to be assessed. Another study described a bacterial factor favoring *S. suis* resistance to human neutrophil-mediated killing: the adenosine synthase of *S. suis* (Ssads) [97]. This enzyme catalyzes the hydrolysis of AMP to adenosine through its 5′-nucleotidase activity. The adenosine then activates the receptor A2aR at the surface of neutrophils, which promote bacterial survival by inhibition of neutrophil functions (see Section 3.6. and Section 3.7. for further details).

A recent article focused on *S. suis* biofilms, a bacterial form of life consisting in grouped bacteria embedded in an extracellular matrix [69]. The authors compared *S. suis* ability to resist neutrophil-mediated killing in its planktonic and biofilm forms and observed that biofilm protects bacteria against killing by neutrophils [69]. Indeed, another study reported that resistance to killing by neutrophils involved the bacterial factor LytR through its participation to biofilm formation and/or nucleotide biosynthesis [92].

Fittipaldi et al. [87] described two surface component modifications that help bacteria to resist neutrophil-mediated killing. Firstly, *S. suis* causes D-alanylation of its lipoteichoic acid, adding positive charges to the negatively charged surface of *S. suis*. It was suggested that this modification interferes with extracellular killing of bacteria, limiting the action of cationic antimicrobial peptides present in the granules of neutrophils [87]. *S. suis* also modifies its peptidoglycan by *N*-deacetylations, known to prevent recognition by host receptors and destruction by the hydrolytic activity of lysozyme in many bacteria [115,116]. The authors found that *S. suis* present low *N*-deacetylation in laboratory growth medium, but in the presence of neutrophils, bacteria overexpress the *pgdA* gene involved in peptidoglycan *N*-deacetylation [86]. They speculated that *pgdA* expression induces an increase in peptidoglycan *N*-deacetylation, preventing killing by the lysozyme of neutrophils [86].

*S. suis* possesses a factor H surface binding protein (Fhb), present at the surface and released by the bacteria, that mediates resistance to killing by human neutrophils [94,98]. Fhb fixes the factor H, a host factor that inhibits the alternative pathway of the complement to avoid an excessive activation [94,98]. This interaction limits complement fixation at the surface of the bacteria [98], and decreases neutrophil clearance by the formation of a large immune complex composed by Fhb, factor H and C3b/C3d [94]. Besides Fhb, other surface components, including the protein Fhbp, are able to recruit factor H in a multifactorial and redundant way [117]. For instance, a triple mutant lacking Fhb, Fhbp and CPS was still able to recruit factor H. In the presence of complement factors, a double mutant lacking both Fhb and Fhbp was similarly killed by pig blood when compared to the wild-type strain [117]. Therefore, more studies are required to clarify the role of factor H recruitment in *S. suis* resistance to neutrophil killing.

*S. suis* could use the presence of fibrinogen in the plasma to its advantage, since it improves bacterial survival to neutrophil-mediated killing [96]. The authors found that the effect of fibrinogen depends on the *S. suis* surface components muramidase-released protein (MRP) and enolase [96], and later described that MRP in a soluble form interacts with fibrinogen promoting bacterial survival in the presence of human neutrophils [93]. Interestingly, fibrinogen also improves *S. suis* biofilm formation [69] and its presence in a whole blood killing assay may enhance bacterial survival [98]. However, if a link exists between those different mechanisms and survival to neutrophil-mediated killing remains to be elucidated.

*S. suis* produces an immunoglobulin M-degrading enzyme (IdeSsuis), also called Mac [89,118]. This enzyme cleaves specifically porcine IgM, annihilating classical complement pathway activation by IgM, and inhibiting neutrophil-mediated killing [89].

A study described that human neutrophils stimulated by *S. suis* present higher amounts of the antimicrobial peptide LL-37 in their secondary granules and release it extracellularly [90]. LL-37 was toxic for *S. suis*, but sublethal doses induced bacterial expression of the aminopeptidase of *S. suis* (ApdS) [90]. ApdS participated in bacterial survival to neutrophil killing, and the authors showed that ApdS cleaves LL-37, protecting bacteria from its bactericidal effect and potentially its chemoattractant properties. The cleavage of LL-37 by ApdS could also reduce neutrophil activation (lower IL-8 production), thus reducing immune cells communication. However, it remains unknown how precisely ApdS promotes bacterial survival [90].

### 3.5. NETs

#### 3.5.1. *S. suis* Induces NET Production by Neutrophils

**Figure 4:**
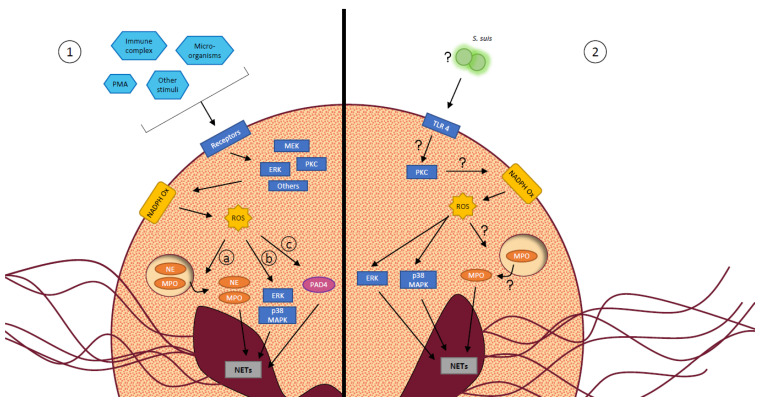
Mechanisms of suicidal NETosis as described using either various stimuli or during *S. suis* stimulation. An arrow represents an activation, a question mark indicates a mechanism that remains to be further confirmed. (**1**) Various stimuli induce neutrophil extracellular trap (NET) formation by neutrophils, including bacteria, phorbol 12-myristate 13-acetate (PMA) and immune complexes. Dependent on the stimuli, the engaged cellular receptors trigger the activation of intracellular pathways (e.g., MEK, ERK or protein kinase C (PKC)), which then lead to the activation of the NADPH oxidase (Ox). This enzyme, pivotal in the formation of NETs, catalyzes reactive oxygen species (ROS) production which is involved in: (a) the release of myeloperoxidase (MPO) and neutrophil elastase (NE) from the granules, which then translocate into the nucleus and participate in chromatin decondensation; (b) the activation of ERK and p38 MAPK that transmit signal for NET formation; and (c) the activation of the peptidyl arginine deiminase 4 (PAD4), an enzyme that citrullinates histones in the nucleus, a feature of NETosis. Only a few of those mechanisms were studied for the induction of NETs by *S. suis.* (**2**) *S. suis*-induced NET formation might depend on Toll-like receptor 4 (TLR4), PKC and NADPH Ox; however, no other receptors or signaling pathways upstream of NADPH Ox were studied. ROS produced by NADPH Ox activates ERK and p38 kinases which participate in NET formation. Despite a role for MPO in NET formation induced by *S. suis*, the intervention of ROS in its release from granules remains to be studied.

NETs are web-like structures composed of decondensed nuclear chromatin extruded outside the neutrophils, coupled with cytosolic and granular proteins [15,16,71]. They take part of the innate immune response by entrapping pathogens, limiting their spread in the host, and by directly killing microorganisms [15,16]. The mechanisms by which neutrophils release NETs is called NETosis. The most described, the suicidal NETosis, includes the following steps: chromatin decondensates in the nucleus, the nuclear envelope breaks down and the chromatin mixes with granular and cytosolic proteins in the cytoplasm. Then, the plasma membrane ruptures leading to cell death and release of the NETs in the environment [119]. To initiate NETosis, cells receive an activation signal through their surface receptors leading to the modulation of various intracellular signaling pathways [16], as described in Figure 4. The vital NETosis, less characterized, happens faster than suicidal NETosis, independently of ROS generation and it does not result in cell death since PMNs remain viable, motile and functional.

Eleven studies reported that neutrophils release NETs in the presence of *S. suis*. NETs were evidenced by confocal microscopy after staining of the DNA, coupled or not with the staining of NET-associated proteins, such as histones [30,54,69,84,91,108,120], neutrophil elastase [70,108] and/or myeloperoxidase [54,75]. Semi-quantitative data were often presented, measuring the number of NET-releasing cells or the area covered with NETs. In addition, NETs were sometime measured in a quantitative manner using PicoGreen^TM^ or Qubit™ dsDNA HS Assay Kits, allowing a quantitative appreciation of free DNA released by neutrophils [69,70,71,84,121]. NETs start to be detectable after 30 min incubation with *S. suis* in the case of pig neutrophils [30], and after 1 to 2 h for mouse neutrophils [70]. Using confocal microscopy, researchers demonstrated *S. suis* entrapment in NETs in vitro [30,69,71,84].

As mentioned, strains from different serotypes and STs could affect immune responses in a different way. For instance, ST28 (serotype 2) and ST1173 (non-typable) strains (isolated from a diseased and a healthy pig, respectively) were unable to induce NET release by porcine neutrophils, while serotype 2 strain 10, a ST1 virulent reference strain, induced strong NET production [120]. Another study reported that neutrophil secretions kill slightly more of a serotype 2 strain of intermediate virulence than two highly ST1 and ST7 virulent strains, even if they activated NET release in the same proportions [71]. This indicates a heterogeneity among *S. suis* strains regarding NET production and their capacity to resist NET killing.

As further discussed in the in vivo section below, NETs are also formed in the brain during meningitis and undergo an ambiguous regulation. Based on these in vivo observations [84,108], models to mimic the dynamic of NET formation after transmigration in the CSF emerged in the literature. The in vitro model of “modified inverse blood–CSF barrier” showed that human neutrophils form NETs after transmigration in the artificial “CSF compartment” and some of them entrapped *S. suis* [84]. Of note, neutrophils migrated to this compartment only when *S. suis* transmigrated first, and NET amounts correlated with that observation (less neutrophils resulted in less NETs) [84]. NET fibers were formed in the presence of *S. suis* in the CSF compartment but host DNAse I inhibited their formation [108].

Only a few studies untangle mechanisms by which *S. suis* stimulates neutrophil release of NETs. A quantitative proteomic analysis revealed an overexpression of certain proteins by neutrophils during *S. suis* stimulation. By inhibiting some of those proteins, the authors identified a role for protein kinase C, NADPH oxidase and myeloperoxidase in the induction of NETs by *S. suis* (Figure 4). Moreover, they found that the matrix metalloproteinase-8 inhibits NET formation by *S. suis*-activated neutrophils [75]. A second study demonstrated that TLR4 recognizes *S. suis* and leads to NET release by neutrophils (Figure 4). This process involves ROS production by NADPH oxidase, and activation of intracellular pathways including p38 mitogen-activated protein kinase (MAPK) and extracellular signal-regulated kinase [70].

#### 3.5.2. *S. suis* Killing by NETs

Since NETs contain granular proteins and histones that potentially kill pathogens, some studies investigated NET ability to kill *S. suis*. In all the experiments, neutrophils were first stimulated with PMA to induce NET release. Then, *S. suis* was added to the cells pretreated with cytochalasin to block phagocytosis, or to the supernatant containing NETs. However, PMA-stimulated neutrophils release not only NETs but also granular proteins and ROS. Indeed, only few studies controlled the specific effect of NETs on bacterial killing using DNAse [69]. Thus, when the authors evocate “killing by NETs” they refer in fact to the killing by “neutrophil secretions”. Porcine neutrophil secretions slightly killed *S. suis* while those of human origin only limited bacterial growth [30]. Using mouse-derived neutrophils, Ma et al. [69] demonstrated that the survival rate of *S. suis* increased when cells were treated with DNAse. Based on that observation, they evaluated killing by neutrophil secretions and demonstrated that supernatants of PMA-treated neutrophils induce 20–25% of *S. suis* killing. However, another study reported absence of *S. suis* killing by murine neutrophil secretions [71].

#### 3.5.3. *S. suis* Defense Mechanisms against NETs

Several bacterial mechanisms could help *S. suis* to resist capture and killing by NETs. *S. suis* possesses two DNAses, a secreted nuclease A (SsnA) and an endonuclease A (EndAsuis). It was shown that SsnA degrades the NETs released by PMA-stimulated human and porcine neutrophils and protects bacteria from killing by human but not porcine neutrophil secretions [30,91]. EndAsuis also degrades NETs produced by human neutrophils but does not protect against killing by neutrophil secretions. Neither SsnA nor EndAsuis affected the number of NET-releasing cells [91] or degraded NETs in the in vitro model of “modified inverse blood–CSF barrier” [84]. Moreover, a study failed to highlight a role for SsnA in pigs in vivo [108]. Thus, the role of *S. suis* DNAses in the pathogenesis of the disease remains unclear and more studies are needed.

Although the CPS constitutes a major virulence factor for *S. suis*, very few studies evaluated its role in NET induction or functions. Two studies evaluated CPS protection against killing by neutrophil secretions (including NETs). In the first one, no role for CPS could be demonstrated, while it protected bacteria in the second study [30,71]. In the latter, the authors also found that CPS allows *S. suis* to evade trapping by NETs [71].

As already mentioned, *S. suis* can also form biofilms. A study demonstrated that the matrix of *S. suis* biofilms inhibits NET formation induced by both bacteria and PMA [69]. Concerning NET ability to kill *S. suis* in its planktonic and biofilm forms, two contradictory results emerged in the study. On one hand, biofilms and free bacterial were killed the same way by “neutrophil secretions”. However, in a classical killing assay, cell treatment with DNAse I indicated that NETs may promote neutrophil-mediated killing. The authors suggested that the inhibition of phagocytosis was more beneficial for the survival of planktonic *S. suis* than the degradation of NETs, while NETs appear to play an important role in biofilm *S. suis* elimination [69].

A study suggested a putative role for *S. suis* cysteine protease ApdS in bacterial resistance to killing by the NETs. Because the technical approach used failed to discriminate between intracellular or extracellular killing, the role of this bacterial protease remains to be confirmed [90].

### 3.6. ROS

Oxygen reduction to water occurs in almost every living cell, as a part of the metabolism. During this process, cells produce many different oxygen-derived intermediates called reactive oxygen species or ROS. They present a single electron on their peripheral layer that give them a very high reactivity potential. ROS were long thought to be deleterious for the cells [14]; however, it is now established that ROS possess antimicrobial properties crucial for defense against pathogens. Phagocytic cells, like neutrophils, produce ROS thanks to a membrane-associated enzyme, the NADPH oxidase. Thus, cell activation leads to NADPH oxidase formation and activation, which convert two molecules of oxygen (O_2_) in two superoxide anions. From this primary ROS, various chemical reactions produce bactericidal molecules such as H_2_O_2_, OH^−^, HOCl, ONOO^−^, etc., which possess different degrees of toxicity [122]. Due to the important diversity of ROS molecules, many different methods exist to reveal oxidative burst (corresponding to the rapid release of ROS by the cells). Some methods, such as those using the fluorescent probes dihydroethidium (DHR123) or dichlorodihydrofluorescein (DCFH-DA), allow measurement of ROS produced, without identification of a specific oxygen-derived intermediate. Other methods measure distinct oxygen radicals or molecules, such as cytochrome c reduction for superoxide anion detection [123]. Only seven studies evaluated ROS production by neutrophils in response to *S. suis*. The majority measured the overall production of ROS as a function of neutrophils [42,63,70,93,97,118]. Only one measured specifically superoxide anion production [74].

In general, an increase of ROS production when neutrophils interact with *S. suis* was reported [70,93,118]. However, the lack of controls, statistical analyses and/or technical details, combined with the variations in the methodology used, do not allow a clear conclusion on *S. suis* effect on ROS production by neutrophils.

ROS were often measured to evaluate the impact of virulence factors on neutrophil functions. Chen et al. [74] demonstrated that *S. suis* supernatant or purified SLY does not affect superoxide anion production by human neutrophils. Another study reported a lack of influence of the *S. suis* IgM protease IdeSsuis [89] on oxidative burst by murine neutrophils [118]. Interestingly, another virulence factor appeared to modify neutrophil oxidative burst: the *S. suis* adenosine synthase Ssads. This enzyme catalyzes AMP transformation to adenosine, whose fixation to its cellular receptor induces a decrease in ROS production [97]. On the other hand, the protein MRP of *S. suis* increases the oxidative burst of human neutrophils. The authors hypothesized that MRP binds to human fibrinogen and activates neutrophils via a β2-integrin-dependant mechanism and suggested that *S. suis* could modulate neutrophil microbicidal functions [93].

Among the studies evocating neutrophil oxidative burst in response to *S. suis*, one was entirely dedicated to untangling the roles of antibodies and complement system on granulocytes’ ability to produce ROS and kill *S. suis* [42]. It was reported that two experimentally infected pigs had different responses towards *S. suis* infection: while one pig presented an important oxidative burst in blood associated with a low bacteremia and high levels of IgG, the other one did not show an early oxidative burst, presented increasing levels of bacteremia and lower levels of IgG. In vitro, the authors demonstrated that granulocytes undergo oxidative burst only in the presence of immune sera, and this participates in *S. suis* killing. The IgG/IgM present in the sera could trigger the oxidative burst in granulocytes as well as activate the complement, which in turn contributes to the oxidative burst axis [42,63]. The study also revealed the importance of the IgM-complement-oxidative burst axis as a mechanism for granulocyte killing of *S. suis* in the blood in the absence of antigen-specific IgG [42].

### 3.7. Degranulation

The presence of granules in the cytoplasm characterizes neutrophils and other granulocytes. These small vesicles contain a variety of proteins including strong antimicrobials. Degranulation occurs when a microbial threat is detected: the granules fuse with the target lipid membrane whose content is released outside the cell or into the phagosome [12]. It exists with different types of granules defined by their protein content and the moment of their formation during the granulopoiesis [124,125]. Azurophilic granules are first formed followed by specific granules, gelatinase granules and finally secretory vesicles. A brief description of their protein content can be found in Table 1. The release of granule content depends on the activating signal intensity: a minimal signal simply induces the release of secretory vesicles, while a stronger signal induces degranulation of gelatinase, specific and ultimately azurophilic granules. Degranulation can be studied by quantifying the granular content in cell supernatants or by analyzing membrane receptors. Indeed, receptors expressed at the membrane of granules—such as CD63, CD11b, CD66b—localize at the cell surface after degranulation.

Only two studies evocated neutrophil degranulation in response to *S. suis*. One aimed to characterize the release of heparin-binding protein (HBP), a granular protein thought to be responsible for vascular leakage in *S. suis*-infected mice [74]. It was shown that human neutrophils stimulated with *S. suis* release HBP and the SLY was identified as a factor involved. Indeed, SLY induced neutrophil degranulation and cells presented membrane blebs, expressed degranulation markers at their surface (CD63, CD11b, CD66b) and released the well-characterized granule proteins elastase and lactoferrin. SLY-dependent release of HBP required Ca^2+^ influx and the cellular pathways p38 MAPK and phosphoinositide 3-kinase. In the second study, the influence of *S. suis* Ssads on neutrophil functions was addressed [97]. It was reported that Ssads decreases CD66 expression by neutrophils (a marker for degranulation) and converts ATP, ADP or AMP to adenosine whose fixation to its receptor might impair degranulation, although no quantitative analyses were presented.

### 3.8. Cytokines

Many different immune cells produce cytokines upon activation, including neutrophils, even if they were long thought to be only effector cells not involved in immunomodulation. Although neutrophils possess less mRNA and produce less cytokines than other immune cells (such as monocytes/macrophages), they are massively recruited during infection and the sum of cytokines released by neutrophils modulates the immune responses [17,126]. Cytokine quantification often consists of measurement of mRNA (RT-qPCR) or the protein itself (ELISA); however, Calzetti et al. [56] insisted on the fact that neutrophil-derived cytokines should be quantified on very pure cell suspensions (more than 99%) in order to avoid contamination by other cytokine-producing cells.

Three studies measured in vitro production of cytokines by neutrophils stimulated with *S. suis*. Murine bone marrow-derived neutrophils primed with LPS produce IL-1β when stimulated with a live serotype 2 *S. suis* strain of high virulence. This production required the caspase 1, which matures IL-1β precursors [72]. The authors suggested a role of SLY, but its implication in neutrophil activation remains to be elucidated. Of note, LPS-primed neutrophils produced TNF-α but *S. suis* did not influence this production [72]. Another study reported that murine neutrophils produce MCP-1 and IL-6 after stimulation with *S. suis* [85]. However, in a porcine model, granulocytes do not produce TNF-α, IL-6 or IL-10 in response to *S. suis* [62]. Indeed, the CPS of *S. suis* prevented its recognition by cells and subsequent production of TNF-α and IL-10, but not IL-6. In these studies, results should be cautiously interpreted since neutrophil purity was lower than 96% or was not measured.

### 3.9. Transmigration of Neutrophils

During *S. suis*-induced meningitis, both bacteria and neutrophils infiltrate the central nervous system (CNS), crossing the blood–brain and/or the blood–CSF barriers [127]. Thus, three studies described in vitro the mechanisms of neutrophil transmigration through the blood–CSF barrier using an inverted transwell filter system to mimic the blood compartment (upper chamber), the blood–CSF barrier (porous filter coated with epithelial cells) and the CSF compartment (lower chamber) [84,108,128]. It was demonstrated that the passage of neutrophils through the epithelial cells occurs by a transcellular way and depends on CD11b/CD18 adhesion molecules. They also observed very few neutrophils crossing spontaneously but a net increase under stimulation with *S. suis* [84,128]. However, it was recently reported that neutrophils transmigrate without stimulation through porcine choroid plexus epithelial cells and *S. suis* did not influence the amount of neutrophils crossing [108]. Moreover, transmigration of neutrophils altered barrier function in a study [128] but not in another [84]. Several cytokines may attract neutrophils to the CNS, but controversial results emerged in the literature. TNF-α favored transmigration in a model [128] but not in another [84], while IL-8 attracted neutrophils in the “CSF compartment” only when the “blood compartment” contained a pure suspension of neutrophils [108,128] but not when using the whole blood [108]. Interestingly, transmigration of neutrophils induced human choroid plexus epithelial cells to produce LL-37, that may attract neutrophils and kill *S. suis* [84]. Considering the few numbers of publications and the lack of consensus between them, further studies are needed to settle which exact mechanisms underline neutrophil transmigration to the brain in response to *S. suis* infection.

## 4. In Vivo Studies

Even if in vitro studies highlight important mechanisms of the host–pathogen interactions and present the advantages of being ethical, reproducible and practical, nothing can replace in vivo experiments to study the immunological events happening in an infected host. Among the 76 studies discussed in this review, 38 evaluated in vivo aspects of neutrophil mobilization, role, functions and regulation during *S. suis* infection.

### 4.1. S. suis-Induced Lesions Contain High Infiltration of Neutrophils

Neutrophil infiltration into the lesions characterizes *S. suis* infection. This was documented at least since 1987 by Sanford et al. who reported neutrophil infiltration in the brain and heart in pigs naturally infected by *S. suis* [10,11]. An early retrospective study reported the clinical signs and lesions of 256 cases of *S. suis* infection in pigs, for the nine most frequently isolated serotypes (serotype 1 to 8 and serotype 1/2) [8]. Most serotypes caused suppurative lesions, meaning that neutrophils accumulated in the organs and formed pus. Since then, neutrophils in the lesions were characterized in various swine studies [9,129,130]. In mouse models, neutrophils also infiltrate both systemic [101,131,132,133,134,135,136] and brain lesions [7,137].

The presence of neutrophils in the lesions allows scoring the importance of the disease induced by *S. suis* since the accumulation of neutrophils witnesses a severe inflammation. Thus, neutrophil infiltration was investigated to measure the protection against *S. suis* in immunization trials in pigs and mice [39,131,135,137,138]; to compare the pathological differences between serotype 2 and 9 in infected pigs [41]; to clarify the importance of virulence factors in the bacterial pathogenesis [89,132]; and to evaluate the severity of the disease caused by the coinfection of *S. suis* and the porcine reproductive and respiratory syndrome virus [139].

### 4.2. Recruitment and Role of Neutrophils during S. suis Systemic Infection

As neutrophils first respond to an infectious threat, different studies aimed to evaluate their mobilization dynamics in the *S. suis*-infected host. In pigs, the common approach consists of routine analysis of blood, although a study employed flow cytometry with swine cell markers [88]. *S. suis* infection caused neutrophil increase in the blood as soon as 2 h post-infection [140] to 4 days post-infection [88,141,142]. Interestingly, two experimental studies reported that neutrophil amounts peak twice in blood during infection, an early peak and a second delayed neutrophil increase whose time-frame varies depending on the study [41,140]. This suggests that important mechanisms regulate neutrophil mobilization during the infection in pigs, but they remain to be elucidated. In mouse models, studies reported neutrophil recruitment in the blood [143,144] and in the peritonea after *S. suis* intraperitoneal infection [69,143]. Interestingly, several studies suggested that the more neutrophil numbers increased, the less bacteria were recovered in the blood [143,144], while others showed that neutrophil mobilization positively correlates with bacteremia levels [140,142]. This suggests that an early mobilization of neutrophils allows *S. suis* clearance but if the infection gets the upper hand, neutrophils are recruited proportionally to bacteremia. Nevertheless, this hypothesis remains to be confirmed.

Several chemokines potentially attract neutrophils during an infection and *S. suis* infection induces the production of many of them, such as CXCL1 (KC), CXCL2 (MIP-2), CCL2 (MCP-1), CCL3 (MIP-1α), CCL4 (MIP-1β) and CCL5 (RANTES) [7,77,85,86,133,145]. In vivo, neutrophil mobilization positively correlated with cytokine/chemokine levels in mouse and porcine models [77,140,145], but only one study established a causality between a cytokine and neutrophil mobilization [144]. Indeed, IL-17A caused neutrophil recruitment in infected mice, despite a minor role in overall *S. suis*-induced inflammation [144].

Neutrophil recruitment during infection is a double-edged sword: if efficient bacterial clearance requires neutrophils, their aggressive mechanisms may cause an exaggerated inflammatory response [146]. A study in a mouse model demonstrated that neutrophils participate in production of pro-inflammatory mediators in plasma, and they promote survival of *S. suis*-infected mice by controlling blood bacterial burden [145]. If neutrophils appear necessary to fight *S. suis* infection, no studies address the neutrophil mechanisms involved, except for the observational correlation between high plasmatic concentration of anti-*S. suis* antibodies, increased granulocyte oxidative burst and controlled bacteraemia in one infected pig [42]. However, those data are limited and the mechanisms by which neutrophils fight *S. suis* in vivo at a systemic level remain an entire field to investigate.

As NETs can also be responsible of exacerbated inflammatory responses [146], different studies documented the presence of NETs in vivo in infected animals. In a farm where pigs died from *S. suis* natural infection, the lungs, kidney and spleen of affected animal(s) contained both bacteria and NETs [120]. In mice, in vivo experiments suggested NET formation in the peritoneum of infected animals [121]. To study the role of the NETs, the authors treated mice with DNAse: they observed greater bacteremia and plasmatic levels of inflammatory meditators, and a lower mouse survival in response to *S. suis* infection. This suggests that NETs are essential for the control of the disease, but it cannot be excluded that DNAse treatment affects other cellular functions, which contribute to the observed effects.

### 4.3. Recruitment and Role of Neutrophils in Central Nervous System Disease Caused by S. suis

Neutrophils seem to be involved not only in the systemic response to *S. suis* infection but also in CNS disease. Indeed, neutrophils infiltrate the brain in the course of the infection and studies recently quantified this infiltration, either after intraperitoneal or intracisternal infection in mouse models [76,77,133,145]. In pigs, only a study highlighted the presence of neutrophils in the brain of animals with meningitis by confocal microscopy [108].

The first clues of the role of neutrophils in CNS disease caused by *S. suis* were given by the study of Auger et al. [145]. They demonstrated that, although neutrophils helped to control brain bacterial burden, they did not contribute to pathological development of meningitis. Interestingly, neutrophil depletion increased levels of pro-inflammatory mediators in the brain. The authors suggested that the higher bacterial burden induced brain resident cells to produce important levels of cytokines in an attempt to recruit neutrophils. In the CNS, neutrophil infiltration would then be a consequence of the inflammation, and not a cause, such as in the systemic infection. Some studies confirmed that neutrophils contribute to the clearance of *S. suis* in the CNS since a higher neutrophil mobilization in the brain is associated with a decrease of bacterial load [76,133].

As NETs may be formed in other streptococcal-induced meningitis and affect bacterial clearance in the brain [147], few studies characterized and investigated the presence of NETs in CNS disease caused by *S. suis* infections. NETs were found in the CSF of naturally or experimentally infected pigs with clinical signs of meningitis [84,108], and *S. suis* was NET entrapped in vivo [84]. The brain tissue of pigs with meningitis also contained NET markers but without the typical fibrous structures [108].

In the brain, some host factors seem to influence NET formation and/or subsistence. For instance, the porcine cathelicidin PR-39 (similar to human IL-77) potentially stabilizes NETs since it might counteract the DNAse activity of CSF samples from *S. suis*-infected pigs [84]; yet this effect remains to be confirmed. Both CSF and choroid plexus of infected pigs with meningitis contained PR-39 and, in the CSF, PR-39 colocalized with NETs [84,108]. Contrary to NET stabilizing factors, the host also possesses its own DNAses in the brain that may disrupt NET fibers. Indeed, DNAse activity increased in the serum and CSF in the early phase of infection, and the DNAse I was detected in the choroid plexus of pigs with meningitis [84,108], which suggest that NET regulation occurs in response to a threat. However, depending on brain compartment, regulation mechanisms differ. In the CSF, the possible stabilization of NETs by PR-39, despite the DNase activity, may prevent bacterial dissemination [84,108]. In the brain tissue, however, the absence of DNA fibers suggests that NET cleavage by host DNAse could prevent damages induced by NETs and/or favor neutrophil-mediated bacterial killing by phagocytosis [108]. The reasons behind this difference of regulation in CSF and brain tissue remain to be elucidated.

### 4.4. Immune Regulation of Neutrophil in Response to S. suis Infection In Vivo

As mentioned previously, neutrophils seem to be finely regulated during the infection and several studies attempted to highlight the mechanisms of neutrophil recruitment, sometimes opening the door for potential treatments against *S. suis*. A study reported that TRIM32 (tripartite motif containing 32) expression, a host regulator of the inflammatory response, reduces the recruitment of neutrophils in the brain during the early course of *S. suis* infection, and thus impairs the clearance of *S. suis* in this organ [133]. Similarly, expression of porcine pentraxin 3, a soluble pattern recognition receptor, limited neutrophil recruitment in the blood of pigs despite a high level of IL-8, a chemokine known to attract neutrophils [142]. Annexin A2, an anti-inflammatory mediator belonging to the glucocorticoids, protects mice from *S. suis* disease likely through reduced inflammation and neutrophil infiltration in the brain [77]. On the other hand, some factors promote neutrophil recruitment such as the triggering receptor expressed on myeloid cells 1 (TREM-1), which participate in bacterial clearance and host survival in the early phase of *S. suis* infection [143,148]. Therefore, the delicate balance between a bactericidal vs. a pathological role of neutrophils, especially in the brain, remains to be further clarified.

Regarding the protective role of neutrophils during the infection, enhancing their recruitment could be an interesting treatment, and an alternative to the use of antibiotics. It was recently observed that *S. suis* infection in a mouse model induces a great production of granulocyte colony-stimulating factor (G-CSF), a cytokine involved in many facets of neutrophil regulation, including their mobilization [149]. Brockmeier et al. [141] exploited G-CSF properties and administrated it in piglets through a replication-defective adenovirus vector. G-CSF massively recruited neutrophils in the blood, which resulted in a reduced bacterial load in organs and an increased survival time of animals. Interestingly, the increased circulation of neutrophils induced by giving G-CSF did not result in pathological effects.

### 4.5. Bacterial Mechanisms against Neutrophils In Vivo

Very few studies investigated the role of *S. suis* virulence factors on neutrophil functions in vivo. Indeed, despite neutrophil mobilization and their role in the control of the infection, *S. suis* still faces neutrophil assault and provokes disease. Investigating the role of the bacterial factor Ssads on gene expression in mouse blood, Dai et al. [150] demonstrated that this factor decreases the expression of genes involved in immune responses, including neutrophil chemoattraction, activation and function. In addition, the SLY may help bacteria to dismantle the neutrophil defense: when comparing in vivo a non-epidemic strain versus an epidemic strain producing four times more SLY, the latter induced more necrosis of peritoneal neutrophils than the non-epidemic strain in *S. suis*-infected mice [101]. It should be noted, however, that SLY is a critical virulence factor for mice but not for pigs, the natural host [4]. Another study also investigated the role of SsnA in vivo in the development of meningitis but it does not appear as a crucial virulence factor [108]. Future studies highlighting how *S. suis* resists neutrophil attacks in vivo may offer very interesting comprehension tools for bacterial pathogenesis.

## 5. Concluding Remarks

Due to policies reducing antimicrobial use in pigs, *S. suis* re-emerges in the porcine industry and represents a zoonotic threat for humans. Thus, there is a necessity to pursue the studies on *S. suis* pathogenesis, especially its interaction with immune cells. Neutrophils first respond to a threat and their intervention seems decisive for the outcome of *S. suis* infection.

**Figure 5:**
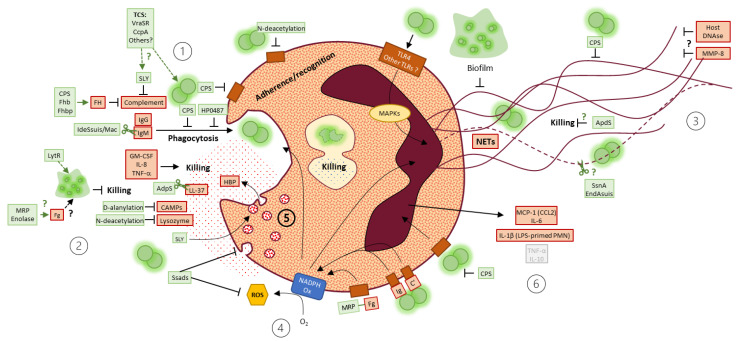
Mechanisms of *S. suis* interference with neutrophil functions (including findings from porcine, murine and human models). In orange are indicated host factors and in green, *S. suis* factors. An arrow represents an activation, a production or an enhancement, while a “T” represents an inhibition/reduction. The scissors symbol refers to a cleavage mechanism. A question mark indicates a mechanism that remains to be further confirmed. (1) The capsular polysaccharide (CPS) of *S. suis* avoids recognition by neutrophils partly because of the negative charge of the CPS. Peptidoglycan modifications by *N*-deacetylation may also limit bacterial recognition. Two-component systems (TCS), such as VraSR and CcpA, can regulate CPS thickness and suilysin (SLY) production. In addition, CPS impairs bacterial phagocytosis/killing by neutrophils, but host complement, IgG and IgM may favor it. However, complement activation may be inhibited by SLY and recruitment of host factor H (FH) at the surface of the bacteria. Indeed, FH is a host factor known to limit complement activation. IgM can be cleaved by IdeSsuis/Mac protease. The surface protein HP0487 may also inhibit phagocytosis. Even if *S. suis* is poorly phagocytized, an intracellular uptake leads to an efficient killing of the bacteria. (2) Cytokines GM-CSF, IL-8 and TNF-α promote bacterial killing. Host LL-37 acts as an antimicrobial but can be cleaved by *S. suis* ApdS. Bacterial *D*-alanylation was suggested to limit the action of cationic antimicrobial peptides (CAMPs), and *N*-deacetylation of the peptidoglycan might limit *S. suis* destruction by lysozyme. Formation of biofilm can be induced by bacterial LytR and presence of host fibrinogen (Fg), and limits neutrophil-mediated killing. Bacterial factors MRP and Enolase adhere to Fg and inhibit killing function of neutrophils. However, if this effect is linked to biofilm formation remains to be determined. (3) *S. suis* induces neutrophil extracellular trap (NET) formation by neutrophils partly through recognition by the Toll-like receptor 4 (TLR4). NET formation depends on MAPK pathways and NADPH oxidase (Ox) activity. However, *S. suis* CPS, DNAses (SsnA and EndAsuis) and ApdS can limit entrapment and/or killing. Host DNAse and matrix metalloproteinase 8 (MMP-8) might release *S. suis* entrapped in NETs or reduce NET formation, respectively (which then would allow bacterial killing by other mechanisms). *S. suis* biofilm can also reduce NET production. (4) Complement (C’), *S. suis*-specific IgG/IgM and MRP-Fg complex activate the NADPH Ox. Bacterial Ssads limits reactive oxygen species (ROS) production by interfering with intracellular pathways. (5) Degranulation results in the release of granular proteins and can be limited by the activity of Ssads. SLY induces release of heparin-binding protein (HBP). (6) Neutrophils produce MCP-1 (CCL2) and IL-6 in response to *S. suis* stimulation. LPS-primed neutrophils also produce IL-1β. CPS limits bacterial recognition by neutrophils and subsequent production of TNF- α and IL-10.

In this review, we first analyzed the literature content and concluded that transposition from an animal model to another, and from a *S. suis* serotype/ST to another should be carefully interpreted and more comparative studies should be performed. We highlighted that viability and purity of neutrophils were neglected in most of the studies, although they critically affect the interpretations of the data. Nevertheless, in vitro studies may provide important comprehension tools on how *S. suis* interacts with neutrophils, highlighting that *S. suis* developed an arsenal of factors to resist neutrophil-mediated killing (Figure 5). However, a gap of knowledge exists on the precise mechanism behind this ability to evade the innate immune system. The poor phagocytosis rate of *S. suis* by neutrophils led researchers to become interested in the “recently” described mechanism of NETs. Often produced in response to *S. suis*, NETs seem a promising mechanism to study in the context of *S. suis* infection. However, other functions such as ROS production, degranulation, cytokine production or transmigration are still understudied. In vivo, the infection provokes a huge systemic mobilization of neutrophils, which seem beneficial to combat the infection. However, more mystery surrounds the role of neutrophils, and particularly the role of NETs, in the development of CNS disease.

Neutrophils are more complex cells than initially described, and recent aspects of their complexity remain to be investigated in the context of *S. suis* infection, such as how the priming affect their functions, what are their dynamics with other cells, how heterogeneous they are, how they age and die, among other aspects. More fundamental studies would improve our understanding of neutrophil dynamics and functions during *S. suis* infections, which could lead to discovering new and better therapeutic tools to control the disease.

## Figures and Tables

**Table 1:** Granules and their protein content.

Granule Name	Protein Content
Azurophilic or primary granules	myeloperoxidase (MPO) elastases (NE) heparin-binding protein (HBP) proteinases defensins
Specific or secondary granules	lactoferrin cathelicidin lysozyme membrane proteins including subunits of the NAPDH oxidase
Gelatinase or tertiary granules	gelatinases lysozyme membrane receptors
Secretory vesicles	HBP membrane proteins including complement receptors
